# When to incorporate point-of-care ultrasound (POCUS) into the initial assessment of acutely ill patients: a pilot crossover study to compare 2 POCUS-assisted simulation protocols

**DOI:** 10.1186/s12947-018-0132-0

**Published:** 2018-09-11

**Authors:** Courtney E. Bennett, Sandhya Samavedam, Namita Jayaprakash, Alexander Kogan, Ognjen Gajic, Hiroshi Sekiguchi

**Affiliations:** 1 Department of Cardiovascular Medicine, Mayo Clinic, 200 First St SW, Rochester, MN 55905 USA; 2 Division of Pulmonary and Critical Care Medicine, Rochester, USA; 3 Division of Health Care Policy and Research, Rochester, USA; 4 Mayo Clinic, Rochester, MN USA; 5 Emergency Services, Rochester, USA; 6 Mayo Clinic Health System in Austin, Austin, MN USA; 7 Division of Pulmonary and Critical Care, Sinai Health System, Chicago, IL USA; 8 Departments of Emergency Medicine and Pulmonary and Critical Care, Henry Ford Hospital, Detroit, MI USA

**Keywords:** Critical illness, Point-of-care, Simulation, Ultrasound

## Abstract

**Background:**

The purpose of this study was to determine the ideal timing for providers to perform point-of-care ultrasound (POCUS) with the least increase in workload.

**Methods:**

We conducted a pilot crossover study to compare 2 POCUS-assisted evaluation protocols for acutely ill patients: sequential (physical examination followed by POCUS) vs parallel (POCUS at the time of physical examination). Participants were randomly assigned to 2 groups according to which POCUS-assisted protocol (sequential vs parallel) was used during simulated scenarios. Subsequently, the groups were crossed over to complete assessment by using the other POCUS-assisted protocol in the same patient scenarios. Providers’ workloads, measured with the National Aeronautics and Space Administration Task Load Index (NASA-TLX) and time to complete patient evaluation, were compared between the 2 protocols.

**Results:**

Seven providers completed 14 assessments (7 sequential and 7 parallel). The median (IQR) total NASA-TLX score was 30 (30–50) in the sequential and 55 (50–65) in the parallel protocol (*P* = .03), which suggests a significantly lower workload in the sequential protocol. When individual components of the NASA-TLX score were evaluated, mental demand and frustration level were significantly lower in the sequential than in the parallel protocol (40 [IQR, 30–60] vs 50 [IQR, 40–70]; *P* = .03 and 25 [IQR, 20–35] vs 60 [IQR, 45–85]; *P* = .02, respectively). The time needed to complete the assessment was similar between the sequential and parallel protocols (8.7 [IQR, 6–9] minutes vs 10.1 [IQR, 7–11] minutes, respectively; *P* = .30).

**Conclusions:**

A sequential POCUS-assisted protocol posed less workload to POCUS operators than the parallel protocol.

## Background

Point-of-care ultrasound (POCUS) is a rapidly evolving diagnostic modality that is performed and interpreted by providers at the bedside [1–7]. An increasing number of reports have shown that POCUS can modify diagnoses, direct further testing, and change medical therapy [8–19]. While the application of POCUS continues to expand, professional societies have published guidelines and recommendations for the use of POCUS specific to their scope of practice. Emergency ultrasound guidelines have identified core emergency ultrasound applications [20], and the Society of Critical Care Medicine recently published guidelines on the use of ultrasonography for critically ill patients [21]. Many clinicians agree that POCUS is complementary to the physical examination. POCUS can be used as a single examination, repeated because of clinical need or deterioration, or used for monitoring physiologic or pathologic changes.

Interestingly, most studies that show clinical utility of POCUS were conducted within 24 h of a patient’s presentation but not always at the time patients were initially assessed. The Focused Assessment with Sonography for Trauma (FAST) examination is one of the very few examples in which POCUS is consistently conducted during the initial resuscitation phase [14, 15, 22]. However, it is still not clear from the original studies if FAST is best conducted in sequence or parallel (or simultaneously) with the primary or secondary survey. In fact, the current guidelines in emergency ultrasound and critical care ultrasonography do not specify the optimal timing of POCUS in relation to the physical examination or other assessments during the initial evaluation of acutely ill patients.

For POCUS to be fully beneficial in patient care, it should be performed and interpreted without interference with the actual workflow during the initial evaluation. Otherwise, POCUS may increase the provider’s workload substantially without adding clinical benefits. We hypothesized that the POCUS-assisted evaluation performed in a sequential manner would pose less workload for the performing providers. To test this hypothesis, we conducted a pilot simulation study to determine the optimal method from the provider’s perspective for integrating POCUS into the initial evaluation of acutely ill patients.

## Methods

### Study setting

We conducted a pilot, unblinded crossover study in which we measured POCUS provider workload to compare 2 POCUS-assisted evaluation approaches: sequential (physical examination followed by multiorgan POCUS) vs parallel (multiorgan POCUS at the time of physical examination). The study was approved by the Mayo Clinic Institutional Review Board (12–007998) and performed from July 13, 2015, through July 26, 2015.

### Study participants

Critical care fellows who participated in the Mayo Clinic institutional POCUS workshop were recruited for the study. The other inclusion criterion was certification with Advanced Cardiac Life Support. There were no exclusion criteria. The fellowship program comprises trainees from Critical Care Medicine and Critical Care/Anesthesia. The programs are 1 to 2 years depending on prior training. The background and training level varied among the participants. All of the fellows participated in the POCUS workshop before recruitment, which is offered in July of each academic year. This course is a full day and includes didactic sessions, hands on learning with standardized patients, and competency testing. Verbal consent was obtained from the study participants.

### Study design

Participants were randomly assigned to group A or group B, according to which POCUS-assisted protocol (sequential vs parallel) was used during simulated patient scenarios. Group A was asked to complete the assessment of patients using the sequential protocol. Group B was asked to complete the same assessment using the parallel protocol. Subsequently, the groups were crossed over to complete assessment using the other POCUS-assisted protocol in the same patient scenarios: group A used the parallel and group B used the sequential protocol. The simulated scenarios were 1) a 30-year-old man with undifferentiated hypotension and 2) a 36-year-old man with acute respiratory distress. POCUS was performed on standardized patients using a VScan device (General Electric, Boston, Massachusetts USA).

**Fig. 1:**
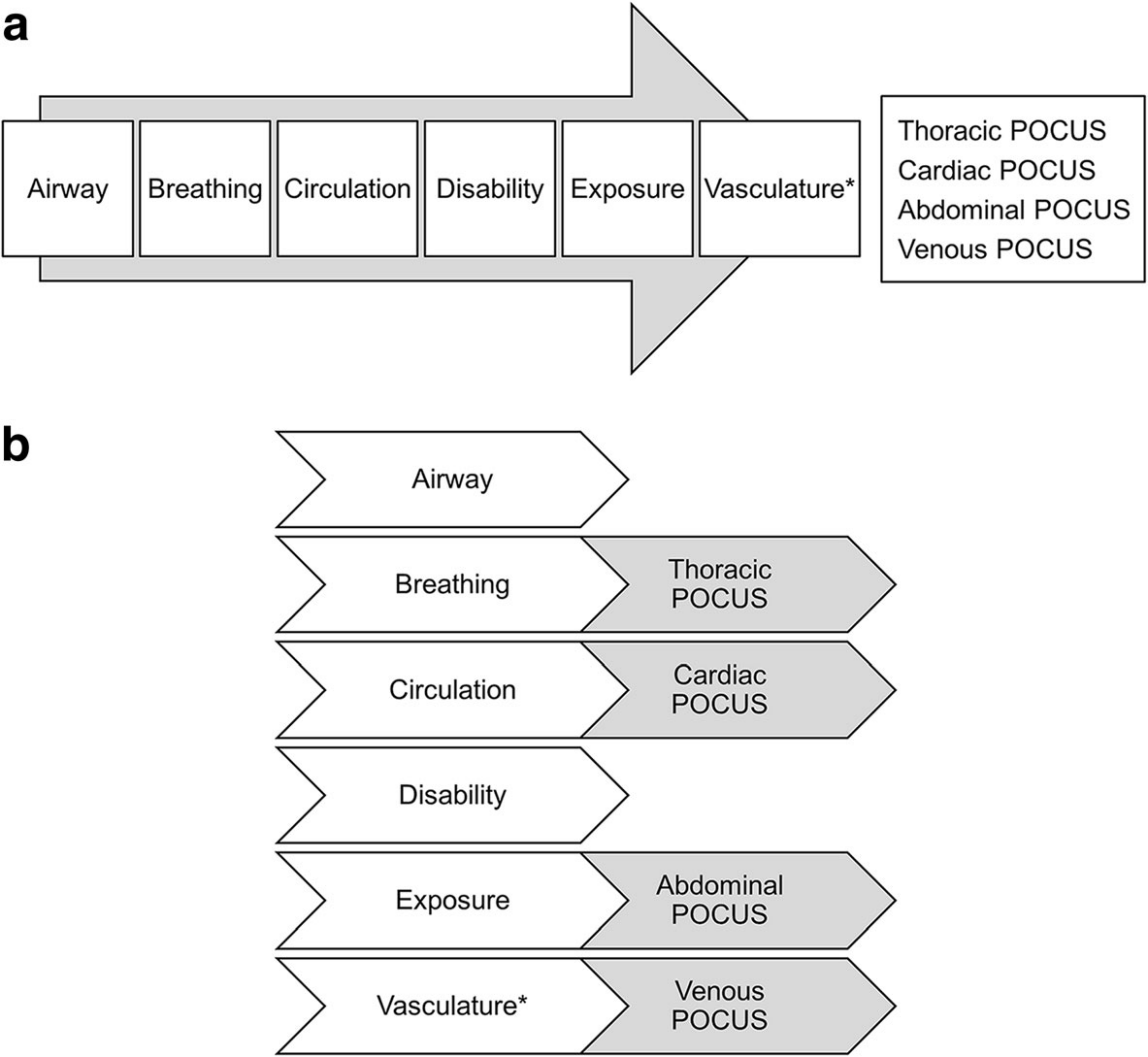
Flowcharts Showing the Sequential and Parallel POCUS Protocols. **a**, In the sequential protocol, the ABCDE (airway, breathing, circulation, disability, and exposure [and vasculature*]) evaluations are completed, and then the system-specific POCUS examinations are performed. **b**, In the parallel protocol, the airway and breathing assessments are completed, followed by the thoracic POCUS; then the circulation assessment, followed by cardiac POCUS; then the disability and exposure assessments, followed by the abdominal POCUS and vasculature* (venous) POCUS. POCUS indicates point-of-care ultrasound. * indicates addition to the ABCDE evaluation

The sequential protocol was performed in the following order (Fig. 1): airway, breathing, circulation, disability, and exposure assessments, followed by thoracic, cardiac, abdominal, and venous POCUS examinations. The parallel protocol was conducted in the following order: airway assessment, breathing assessment, then thoracic POCUS; circulation assessment, then cardiac POCUS; disability assessment, exposure assessment, then abdominal POCUS and venous POCUS (vasculature). The POCUS examinations had 10 required ultrasound examination points and 6 optional points, for a total of 16 potential points. The examination sites for the POCUS protocol are shown in Table 1. Participants were asked to complete a National Aeronautics and Space Administration Task Load Index (NASA-TLX) survey after they finished the 2 simulated patient assessments [13].

**Table 1:** Ultrasound Transducer Application Sites in the POCUS Protocol

POCUS Type by Application Site, No.	Transducer Application Site
Thoracic, 6	Upper anterior chest
	Lower anterior chest
	Upper lateral chest (3 application sites per hemithorax)
Cardiac, 1 or 3^a^	Subcostal, including inferior vena cava
	Parasternal^a^
	Apical^a^
Abdomen, 3	Upper right flank
	Upper left flank
	Pelvis
Vasculature, 4^a^	Inguinal vein^a^
	Popliteal vein^a^ (2 application sites per lower extremity)

Abbreviation: POCUS, point-of-care ultrasound

^a^Optional based on clinical indication

### Outcome variables

The NASA-TLX survey was used to measure perceived workload as the primary outcome. NASA-TLX is an integrated measure of overall workload [13]. It consists of 6 domains that represent clustered independent variables: mental demand, physical demand, temporal demand, performance, effort, and frustration [12]. Scores in each domain and overall raw NASA-TLX scores were collected from participants after they completed the 2 patient scenarios. Time to complete the assessment was also recorded. Qualitative feedback from participants about 2 POCUS-assisted protocols was also collected in a survey format.

### Statistical analysis

Descriptive statistical terms, such as median and interquartile range (IQR) were used to summarize the outcome variables. Variables were compared between sequential and parallel POCUS-assisted protocols using the Wilcoxon signed rank test. JMP 10.0 software (SAS Institute Inc., Cary, North Carolina USA) was used for the statistical analysis. Because this was a pilot study, sample size calculation was not performed. A *P* value of <.05 was considered significant.

## Results

Seven critical care fellows were enrolled in the study: 4 fellows were assigned to group A (sequential then parallel POCUS-assisted protocol); 3 fellows to group B (parallel then sequential POCUS-assisted protocol). There were 3 trainees from Critical Care Medicine (all postgraduate year [PGY] 4), 1 trainee from critical care emergency medicine (included in the Critical Care Medicine program; PGY 6), and 3 trainees from Critical Care/Anesthesia (all PGY 5). A total of 7 sets of NASA-TLX scores, time to complete patient assessments, and participant surveys were collected for each POCUS-assisted protocol (sequential and parallel). The median NASA-TLX score and time to complete assessment are shown in Table 2. We observed a significant difference in the median (IQR) total NASA-TLX scores between the sequential and the parallel protocols (30 [30–50] versus 55 [50–65], respectively; *P* = .03), with the sequential protocol being associated with a lower provider workload. When evaluating the individual components of the NASA-TLX scores, we observed a significantly lower median (IQR) mental demand and frustration level for the sequential protocol than the parallel protocol: 40 (30–60) vs 50 (40–70) (*P* = .03) and 25 (20–35) vs 60 (45–85) (*P* = .02), respectively. The scores for the other 4 scales (physical demand, temporal demand, performance, and effort) were not significantly different between the 2 protocols.

**Table 2:** Comparison Between Sequential and Parallel POCUS-assisted Protocols in NASA-TLX Score and Time to Complete Assessment

Metric	Sequential, Median (IQR)	Parallel, Median (IQR)	*P* Value
NASA-TLX score (of 100)			
Overall	30 (30–50)	55 (50–65)	.03
Mental	40 (30–60)	50 (40–70)	.03
Physical	30 (25–50)	60 (60–70)	.06
Temporal	50 (30–55)	50 (30–60)	.81
Performance	35 (30–60)	45 (40–70)	.06
Effort	50 (30–70)	65 (45–75)	.28
Frustration	25 (20–35)	60 (45–85)	.02
Time to complete assessment, min	8.7 (6–9)	10.1 (7–11)	.30

Abbreviations: POCUS, point-of-care ultrasound; NASA-TLX, National Aeronautics and Space Administration Task Load Index

Fellows commented that maneuvering between the physical examinations with a stethoscope and then an ultrasound transducer in the parallel POCUS-assisted protocol was distracting and more frustrating than completing the physical examination and then performing the POCUS examination in the sequential protocol. Fellows also suggested that the time needed to remove the ultrasound gel between examination components prolonged the time needed to complete the examination. Despite these perceptions, the median (IQR) time needed to complete patient scenarios was not significantly different between the 2 protocols: 8.7 (6–9) minutes in the sequential and 10.1 (7–11) minutes in the parallel protocol (*P* = .30).

## Discussion

Our study demonstrated that the sequential protocol posed less workload for POCUS operators than the parallel protocol, although the time to complete assessments was not significantly different between the 2 protocols. Mental demand and frustration level were significantly lower in the sequential protocol. To our knowledge, this pilot study is the first to assess the most efficient timing for integrating POCUS with the physical examination by evaluating workload perceived by the POCUS operators.

As POCUS is a relatively new diagnostic and procedural modality, previous literature has focused on investigating its emerging indications and clinical utility, and emphasis was more on demonstrating its advantage over physical examination alone [1–11]. Therefore, less attention was paid to the timing of POCUS and how POCUS is best incorporated in the clinical workflow. For instance, a study in an emergency department found that POCUS conducted immediately after the recognition of undifferentiated hypotension decreased physicians’ diagnostic uncertainty and allowed them to modify treatment plans and determine the need for and place of admission [18]. However, in this study, the physician performing POCUS was not the clinician performing the history and physical examination. Another study performed in an emergency department also showed that POCUS reduced the number of viable diagnoses when it was performed early [16]. In this study, the ultrasound examination was performed after a history, physical examination, and “standard care interventions” were performed. Therefore, in both of these studies, the ultrasound examination was performed after the history and physical examination had been completed. Neither study assessed the integration of POCUS into the initial evaluation phase or the provider’s workload associated with introduction of the POCUS examination.

Studies conducted in intensive care units [10, 11] have had similar issues to those conducted in emergency departments. A study evaluating the role of cardiac POCUS in patients with shock showed that cardiac POCUS influenced fluid and inotropic therapy and that its use was associated with decreased mortality [9]. In this study, cardiac POCUS was performed between 7.5 to 15 h from the time the patient arrived in the emergency department and not at the initial evaluation. Another study evaluated the use of POCUS for determining the cause of pulmonary edema in patients in the intensive care unit [19]. The patients who underwent POCUS immediately after pulmonary edema was diagnosed had the cause identified significantly sooner than a control group. In this study, POCUS was performed after the initial history taking and physical examination, and no discussion on the best timing of POCUS was made [19]. The same limitation applies to a study about the “ICU-sound” protocol, a “head-to-toe” POCUS examination within 12 h of admission that resulted in a modified admitting diagnosis in 26% of the cases [8].

Current guidelines in emergency ultrasound and critical care ultrasonography do not specify the optimal timing of POCUS in relation to the physical examination or other workflow during the initial evaluation of critically ill patients. POCUS is well known for being operator dependent in image acquisition and interpretation. Furthermore, integration of POCUS-obtained information into clinical decision-making is also highly operator dependent. Our study results show that a sequential approach is more practical than a parallel approach for maximizing the benefit of POCUS in patient care without causing a substantial workload for providers.

This pilot study has several limitations. Our sample size was very small. However, we did observe a significant difference in several metrics related to the POCUS operator’s workload. The patient scenarios (undifferentiated hypotension and respiratory distress) were arbitrary, although they are among the most common patient presentations in intensive care units. The 2 protocols were compared in a hypothetical situation, in which the primary provider conducted the initial evaluation and resuscitation. It is not clear if the same conclusion can be drawn regarding a preferred POCUS-assisted protocol in a situation where a provider acts as a team leader while other team members conduct history taking, physical examination, and the POCUS examination.

## Conclusion

In summary, a sequential POCUS-assisted protocol posed less workload to POCUS operators than a parallel protocol. For POCUS to be fully beneficial in clinical care, it needs to be integrated into current workflow without a substantial burden to providers. Our study supports the use of a sequential approach when POCUS is incorporated into the initial resuscitation and evaluation phase.

## Abbreviations

FAST: Focused Assessment with Sonography for Trauma
IQR: Interquartile range
NASA-TLX: National Aeronautics and Space Administration Task Load Index
PGY: Postgraduate year
POCUS: Point-of-care ultrasound

## References

[1] Ferrada P, Vanguri P, Anand RJ, Whelan J, Duane T, Aboutanos M, Malhotra A, Ivatury R (2013). A, B, C, D, echo: limited transthoracic echocardiogram is a useful tool to guide therapy for hypotension in the trauma bay--a pilot study. J Trauma Acute Care Surg.

[2] Hollister N, Bond R, Donovan A, Nicholls B (2011). Saved by focused echo evaluation in resuscitation. Emerg Med J.

[3] Gunst M, Sperry J, Ghaemmaghami V, O'Keeffe T, Friese R, Frankel H (2008). Bedside echocardiographic assessment for trauma/critical care: the BEAT exam. J Am Coll Surg.

[4] Pershad J, Myers S, Plouman C, Rosson C, Elam K, Wan J, Chin T (2004). Bedside limited echocardiography by the emergency physician is accurate during evaluation of the critically ill patient. Pediatrics.

[5] Copetti R, Soldati G, Copetti P (2008). Chest sonography: a useful tool to differentiate acute cardiogenic pulmonary edema from acute respiratory distress syndrome. Cardiovasc Ultrasound.

[6] Chin EJ, Chan CH, Mortazavi R, Anderson CL, Kahn CA, Summers S, Fox JC (2013). A pilot study examining the viability of a prehospital assessment with UltraSound for emergencies (PAUSE) protocol. J Emerg Med.

[7] Bagheri-Hariri S, Yekesadat M, Farahmand S, Arbab M, Sedaghat M, Shahlafar N, Takzare A, Seyedhossieni-Davarani S, Nejati A (2015). The impact of using RUSH protocol for diagnosing the type of unknown shock in the emergency department. Emerg Radiol.

[8] Manno E, Navarra M, Faccio L, Motevallian M, Bertolaccini L, Mfochive A, Pesce M, Evangelista A (2012). Deep impact of ultrasound in the intensive care unit: the "ICU-sound" protocol. Anesthesiology.

[9] Kanji HD, McCallum J, Sirounis D, MacRedmond R, Moss R, Boyd JH (2014). Limited echocardiography-guided therapy in subacute shock is associated with change in management and improved outcomes. J Crit Care.

[10] Zieleskiewicz L, Muller L, Lakhal K, Meresse Z, Arbelot C, Bertrand PM, Bouhemad B, Cholley B, Demory D, Duperret S (2015). Point-of-care ultrasound in intensive care units: assessment of 1073 procedures in a multicentric, prospective, observational study. Intensive Care Med.

[11] Bernier-Jean A, Albert M, Shiloh AL, Eisen LA, Williamson D, Beaulieu Y (2017). The diagnostic and therapeutic impact of point-of-care ultrasonography in the intensive care unit. J Intensive Care Med.

[12] Hart SG (2006). NASA-task load index (NASA-TLX); 20 years later. Proceedings of the Human Factors and Ergonomics Society Annual Meeting.

[13] Hart SG, Staveland LE (1988). Development of NASA-TLX (task load index): results of empirical and theoretical research. Adv Psychol.

[14] Arrillaga A, Graham R, York JW, Miller RS (1999). Increased efficiency and cost-effectiveness in the evaluation of the blunt abdominal trauma patient with the use of ultrasound. Am Surg.

[15] Rose JS, Levitt MA, Porter J, Hutson A, Greenholtz J, Nobay F, Hilty W (2001). Does the presence of ultrasound really affect computed tomographic scan use? A prospective randomized trial of ultrasound in trauma. J Trauma.

[16] Jones AE, Tayal VS, Sullivan DM, Kline JA (2004). Randomized, controlled trial of immediate versus delayed goal-directed ultrasound to identify the cause of nontraumatic hypotension in emergency department patients. Crit Care Med.

[17] Scalea TM, Rodriguez A, Chiu WC, Brenneman FD, Fallon WF, Kato K, McKenney MG, Nerlich ML, Ochsner MG, Yoshii H (1999). Focused assessment with sonography for trauma (FAST): results from an international consensus conference. J Trauma.

[18] Shokoohi H, Boniface KS, Pourmand A, Liu YT, Davison DL, Hawkins KD, Buhumaid RE, Salimian M, Yadav K (2015). Bedside ultrasound reduces diagnostic uncertainty and guides resuscitation in patients with undifferentiated hypotension. Crit Care Med.

[19] Wang XT, Liu DW, Zhang HM, Chai WZ (2014). Integrated cardiopulmonary sonography: a useful tool for assessment of acute pulmonary edema in the intensive care unit. J Ultrasound Med.

[20] American College of Emergency Physicians (2009). Emergency ultrasound guidelines. Ann Emerg Med.

[21] Frankel HL, Kirkpatrick AW, Elbarbary M, Blaivas M, Desai H, Evans D, Summerfield DT, Slonim A, Breitkreutz R, Price S (2015). Guidelines for the appropriate use of bedside general and cardiac ultrasonography in the evaluation of critically ill patients-part I: general ultrasonography. Crit Care Med.

[22] Melniker LA, Leibner E, McKenney MG, Lopez P, Briggs WM, Mancuso CA (2006). Randomized controlled clinical trial of point-of-care, limited ultrasonography for trauma in the emergency department: the first sonography outcomes assessment program trial. Ann Emerg Med.

